# Analysis of ecotoxic influence of waste from the biomass gasification process

**DOI:** 10.1007/s11356-017-9011-8

**Published:** 2017-05-10

**Authors:** Małgorzata Hawrot-Paw, Adam Koniuszy, Małgorzata Mikiciuk, Monika Izwikow, Tomasz Stawicki, Paweł Sędłak

**Affiliations:** 1 Department of Agrotechnical Systems Engineering, West Pomeranian University of Technology, Papieża Pawła VI 1, 71-459 Szczecin, Poland; 2 Department of Plant Physiology and Biochemistry, West Pomeranian University of Technology, Słowackiego 17, 71-434 Szczecin, Poland

**Keywords:** Plant, Soil, Diesel, Biodiesel, Microorganisms, Biomass, Gasification

## Abstract

The purpose of this research was evaluation of the effect of soil contamination with waste coming from biomass gasification on chosen indicators of its biological activity, growth and development of spring barley, and change of physiological parameters of the plant. Chromatographic content and basic rheological parameters of the substances under research were also analyzed. Liquid wastes, tar, and mixture of tar and engine oil were introduced to the soil in the amount of 100 mg kg^−1^ DM soil. Based on the conducted research, it was ascertained that the changes in the number and activity of soil microorganisms were determined by the type of waste and its dose. Individual groups of microorganisms showed different sensitivity to the presence of pollution; however, the impact of tar and engine oil mixture was generally more disadvantageous. Presence of contaminants in the soil limited the growth of roots and aboveground parts of spring barley, especially when the dose was 10,000 mg kg^−1^ DM soil. The unfavorable impact of waste on photosynthesis efficiency on assimilation pigment synthesis and water content in the plant was recorded.

## Introduction

Biomass has always been the main source of energy for people and still is the most widespread of its forms (McKendry 2002). Annual crop of biomass is ca 220 milliard tones (Ren et al. 2009), and it is estimated that it meets world energy needs in 10–14% (McKendry 2002). Currently, new alternative energy sources are being sought for as well as new technologies of its acquisition. Biomass is not only the green herbaceous material but also waste coming from agriculture and forestry. The ability of their energy utilization corresponds with the principle of balanced development, inscribing itself in so-called circular economy.

There is a number of methods of biomass conversion (thermochemical, biochemical, physicochemical), which allow the release of inherent chemical energy and obtaining this way thermal energy or biofuels. Among the thermochemical methods can be found among others burning, gasification, pyrolysis, and fluidization (Tchabda and Pisupati 2014). Gasification is one of the oldest technologies, which involves the change of fuel content by heating and chemical reaction with oxidant in the conditions of their limited access (which means lack of oxygen relatively to the stoichiometric requirement). In this process, so-called synthesized gas, which consists of 18–20% of CO, 18–20% of H_2_, 8–10% of CO_2_, and 2–3% CH_4_ (Bridgwater 2003, Larsen et al. 2003), is primarily formed and furthermore ash and tar as the result of incomplete biomass conversion (Kumar et al. 2009). One of the components formed in the process are hydrocarbons including polycyclic aromatic hydrocarbons. They are a durable organic waste, which is easily tied in the surface layer of the soil (Wilcke 2007), they can accumulate in plants, and in this way, they can incorporate in the food chain, becoming a risk for human health and life. Physiochemical methods used to control this type of pollution often increase the problem instead of decreasing or eliminating it (Umanu et al. 2013). Analytical methods are not sufficiently sensitive elements of environmental danger assessment thus the need of monitoring it with living organisms. Especial role in this range is played by microorganisms (Logar and Vodownik 2007) and plants (Hawrot and Nowak 2005).

Gasification of biomass is very interesting alternative for bioenergy production. Syngas can be used in electricity and steam generation, transportation fuels, or hydrogen production. This method helps to reduce dependence of fossil fuels, and it is therefore called environment-friendly technology (Madadian et al. 2014), but we cannot forget that the process of gasification generates by-products, wastes. Their properties depend on the feedstock and the gasification process conditions. The wastes could be toxic or hazardous even if present in amounts that are not detectable by analytical methods. Chemical approach does not allow to identify all the compounds. We need more sensitive indicators of possible pollution customized to the type of contamination. The feasibility of using ecotoxicological tests for this purpose has been determined in this study. The scope of work also included the determination of modification effect of gasification process (filter of combustion engines in the installation was filled or not with engine oil) on the toxicity of wastes. It is very important to know how they will affect the environment because their presence may entail a lot of problems. The applied tests contained biological indicators belonging to various groups—microorganisms and plants. These tests could inform about the ability to restore soils their original function as a living environment for other organisms and could also be used to monitor the effectiveness of remedial treatment.

## Materials and methods

### Chemicals samples and analysis

Material for this research has been obtained from the technological process including gasification of wooden chips in order to obtain wood distillation gas, cleaning of the wood distillation gas with the use of filter, and generating electrical energy and heat by use of combustion engine. During this process, ash is produced not only as the main solid waste but also as liquid waste, which is the mixture of water and tarry impurities, is produced. Water and light tar substances are outdropped in the cooler of the pilot installation; however, the residual tares are separated in centrifugal filters of combustion engines, where the material used in research came from. In the first case, the bottom of the filter was filled with engine oil necessary for its proper work (whirling stream of crude gas glances off from the surface of oil, to which impurities with bigger mass attach). After ca 12 h of work of the system, the initial volume of the engine oil (100 cm^3^) increased to 500 cm^3^ due to tar substances, which were precipitated in this filter. In the second case, the bottom of the filter was filled with engine oil due to the assumption that tar substances separated in the cyclone part of the filter, no matter what, will flow to its bottom. After 12 h of work, their volume was 350 cm^3^. In this way, two types of research material have been obtained: tar with engine oil in the proportion 4:1 (A) and pure tar (B).

Analysis of the content of examined substances was conducted in Institute of Organic Chemical Technology of West Pomeranian University of Technology in Szczecin. Samples of the material were dissolved in DMF (dimethyl formamide), and in addition, dissolving was supported with ultrasounds. Conditions of conducted analysis: gas chromatograph Trace 2000 GC produced by Thermo, column: TR-WAX produced by Thermo; (30 m long, internal diameter 0.25 mm, film thickness 0.5 μm), carrier gas: helium (constant flow), flow rate of carrier gas: 1.8 cm^3^ min^−1^; mass detector MS Voyager produced by Finnigan (quadrupole); ionization method: electronic; membrane feeder with distribution: split/splitless (SSL); registration and integration: Xcalibur v. 1.2 computer system produced by Finnigan Corporation.

Basic rheological research of tar materials including analysis of dynamic viscosity in temperature range of 5–90 °C was made. Viscometer Brookfield DV-II+ was used in the research. The sample temperature in the viscometer was kept by a thermostat. The first measurement of dynamic viscosity was started from the lowest temperature and every next at 5 °C. For each measurement point, the temperature was kept by 10 min to stabilize and measure. Torque of roller in a cylinder filled with samples oil was measured. The resistance of the rotating roller was the scale of the dynamic viscosity. For the adopted measuring temperatures, three times repetition was done based on which the average values of dynamic viscosity ware estimated and next used to determine the charts of viscosity changes in temperature function of sample *ῃ* = f(T).

### Soil

Soil was collected from the Gumieniecka Plain (Poland, 53° 40′ N, 14° 47′ E). Soil samples were taken from 0 to 15 cm of arable-humic horizon. They were air-dried and crushed to pass through a 2-mm mesh sieve. The soil belongs to chernozems, regarding to the particle size composition, represents sandy loam. The basic characteristics of this soil are presented in Table 1.

**Table 1:** Physiochemical properties of the soil

Percentage of fraction			C	N	P	K	Mg	pH_KCl_
2.0–0.05	0.05–0.002	<0.002	g kg^−1^ DM soil		mg kg^−1^ DM soil			
58.4	38.7	2.9	21	1.5	120	89	94	6.80

### Experimental design and biological analysis

Biological research was conducted in loamy sand within two experiments. In the first experiment, the impact of waste from the gasified biomass on soil microorganisms was determined; however, in the second experiment, the evaluated subject was their influence on plants. Within the no. 1 laboratory experiment, 1-kg soil samples after determination of current soil moisture by drier-weight method were brought to 50% capillary water capacity. Next, mixture of tar and engine oil (objects A) or pure tar (objects B) in three doses: 1–100, 2–1000, and 3–10,000 mg kg^−1^ DM soil was introduced into the soil. Control object (C) was unpolluted soil. The experiment involved three repetitions for each variant. Samples (1 kg of soil with pollution) were placed in polyethylene containers (2.5 l capacity) and stored for the period of 28 days in the temperature of 20 °C. In the appropriate dates (1, 14, 28 days of incubation), planned microbiological analysis including determination of dynamics of changes in number and activity of soil microorganisms was made. The number of microorganisms was determined by soil-diluted inoculation method, including bacteria on Bunt and Rovira (1955) medium, fungi on Martin (1950) medium, and the order actinobacteria on Cyganov and Žukov (1964) medium, respectively, after 3, 5, and 7 days of incubation in the temperature of 20 °C. Furthermore, the number of copiotrophic microorganisms on NB medium and oligotrophic microorganisms on DNB medium, respectively, after 7 and 14 days of incubation in the temperature of 28 °C (Ohta and Hattori 1980) was determined. Based on the number of those microorganisms, the O:C indicator, which is one of the biological soil balance (Weyman-Kaczmarkowa 1995) indicators and informs about directions of organic matter conversion, was determined. The results (average from three dates of analysis) were presented as CFU (colonies forming units) recalculated for 1 g DM soil. Activity of soil microflora polluted with examined substances was determined based on measurement of biomass of living organism content by SIR (substrate-induced respiration) method (Anderson and Domsch 1978). Soil samples (10 g) were enriched with additional 0.3 g carbon source in the form of glucose and talc mixture (ratio 1:5) and then placed in measuring columns of Ultragas U4S analyzer. The carbon dioxide evolution was measured after 3 h. Microbial biomass was calculated using the equation:

$$ X=40.04\times Y+0.37, $$

where

*X*: the amount of C contained in the microbial biomass [mg C 100 g^−1^ DM soil]
*Y*: maximum initial production of CO_2_ [cm^3^ h^−1^ 1 g^−1^ DM soil].

All microbiological designations were made in three repetitions.

Laboratory experiment no. 2 which aimed at evaluation of phytotoxic impact of waste from gasification on growth and development of spring barley conducted with pot method according to the standard (PN-ISO 11269-2-2001). Three pots for each experimental combination were prepared. Ten seeds of spring barley were sown in each pot. Basic biometrical parameters—the height of the part aboveground and length of the root (in mm) were determined for all plants from each pot.

After completing the pot experiment, the plant matter was the subject of physiological analysis. Content of chlorophyll “a”, “b” and total chlorophyll in leaves was determined with Arnon et al. methods (1956) in Lichtenthaler and Wellburn modification (1983), however, the content of carotenoids with Hager and Mayer-Berthenrath method (1966). For the estimation of assimilation pigment content, a known mass of leaf (about 0.05 g) was homogenized in 10 cm^3^ of 80% acetone. The homogenate was centrifuged at 2500 g for 10 min. Optical density of the samples was determined spectrophotometrically with wave length *λ* = 440, 645, and 663 nm using a spectrophotometer (Marcel Mini). Content of assimilation pigments was expressed in mg kg^−1^ fresh mass of the plant. Index of relative water content in leaves (RWC) was determined according to Yamasaki and Dillenburg (1999). Leaf material was weighed to determine fresh weight and placed in distilled water for 24 h, and then turgid weight was recorded. Finally, the samples were dried in an oven at 80 °C for 48 h and the dry weights were recorded. RWC was calculated as:

$$ \mathrm{RWC}=\frac{\left(\mathrm{fresh}\ \mathrm{weight}-\mathrm{dry}\ \mathrm{weight}\right)\ }{\left(\mathrm{turgid}\ \mathrm{weight}-\mathrm{dry}\ \mathrm{weight}\right)}\times 100 $$

Determination was done in four repetitions.

Using spectrofluorimeter Handy PEA (Hansatech) according to standard procedure of the device, after 20 min adaptation of leaves to darkness, on six leaves from each experimental variant, measured and calculated were following parameters of fluorescence induction of chlorophyll: F_V_/F_M_—maximum, potential efficiency of photochemical reaction in PS II determined after dark room adaptation, after acceptors reduction in PS II (Bolhár-Nordenkampf and Öquist 1993), T_FM_—time of chlorophyll fluorescence increase from the beginning of measurement to reaching maximum (F_M_), PI—vitality indicator PS II, which concerns general vitality of this system, and area—pool of reduced plastoquinone electron acceptors.

### Statistical analysis

The results of this research have been statistically analyzed using program Statistica ver. 12.0 made by StaSoft Poland. Significance of differences, based on which the average for interaction was allocated to homogeneous groups, was determined by Duncan test on the level of significance *α* = 0.05.

## Results and discussion

**Fig. 1:**
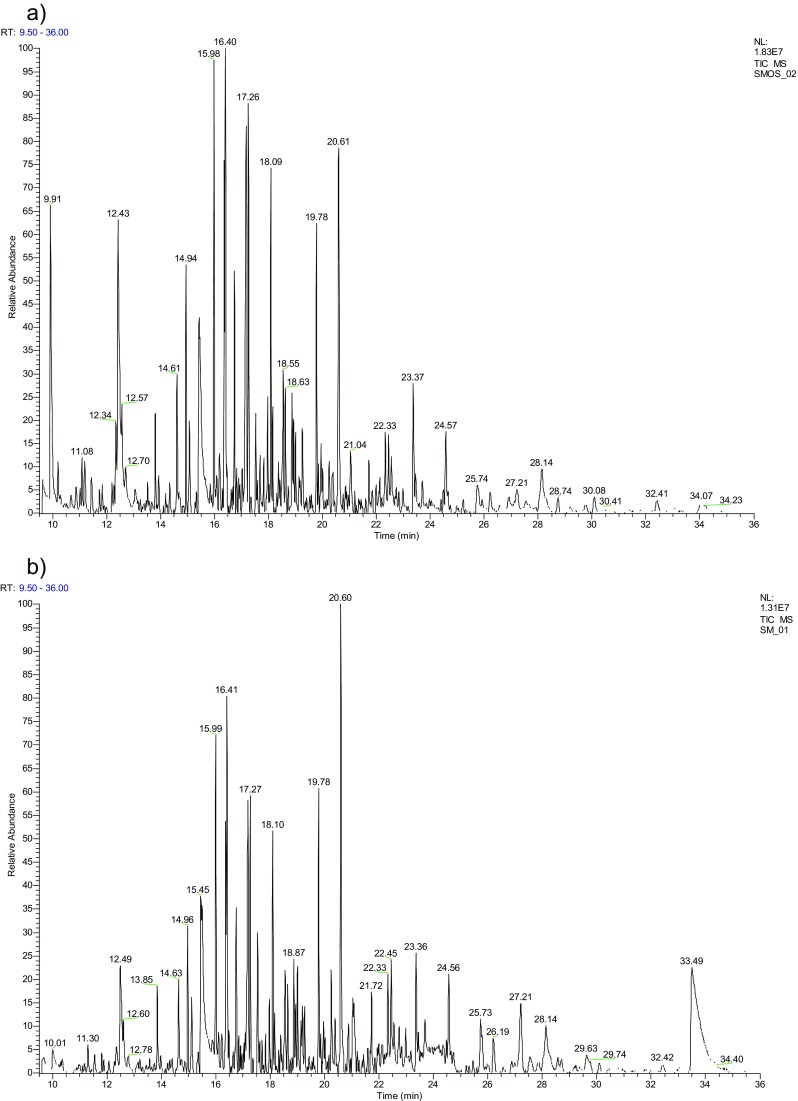
Chromatographic content of tar and engine oil mixture (**a**) and tar (**b**) from the gasification process of biomass

The results of chromatographic analysis of the substances under research are presented in Fig. 1. In the content of both products, however, with slight difference in content, presence of ingredients typical for tar coming from biomass gasification, among other heterocyclic compounds (phenol, creosols), monochromatic and polycyclic hydrocarbons (Coll et al. 2001), was found. Also acetic acid was noted among the content of tar and engine oil mixture.

**Fig. 2:**
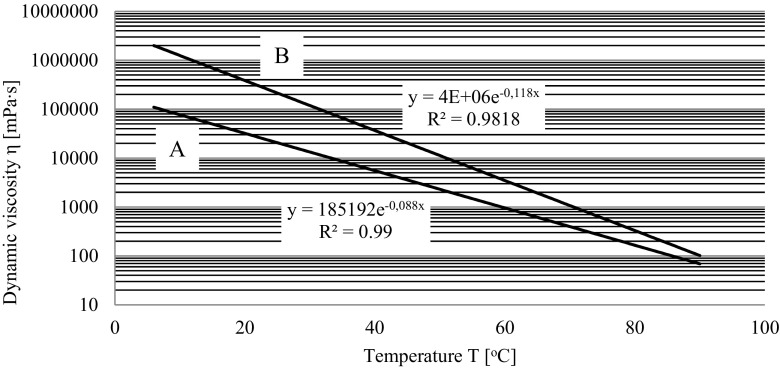
Dynamic viscosity of the tar and engine oil mixture (*A*) and tar (*B*) as function of temperature

In the research concerning dynamic viscosity of the analyzed samples, a very wide range of value variation of this parameter in the assumed range of measuring temperature (Fig. 2) was ascertained. Determined theoretical models of viscosity changes, being the function of temperature, taking into consideration their matching to empiric data (*R* ^2^ near 1.0) give base to estimating differences in dynamic viscosity of substances under research, with previously established comparative temperature. For temperature of 20 °C, meeting thermal conditions of the conducted biological research was established that 25% share of engine oil in tar influenced the decrease of this mixture viscosity by 350,000 mPas, that is, by more than 91% compared to the tar sample not including engine oil. Viscosity of impurities influences their activity in soil, process of soil sorption, and ability to move and also decides about bioavailability. Presence of engine oil in the mixture could have caused disadvantageous changes in soil microstructure (Nazir 2011), thus indirectly influence in negative way on its biological life.

**Fig. 3:**
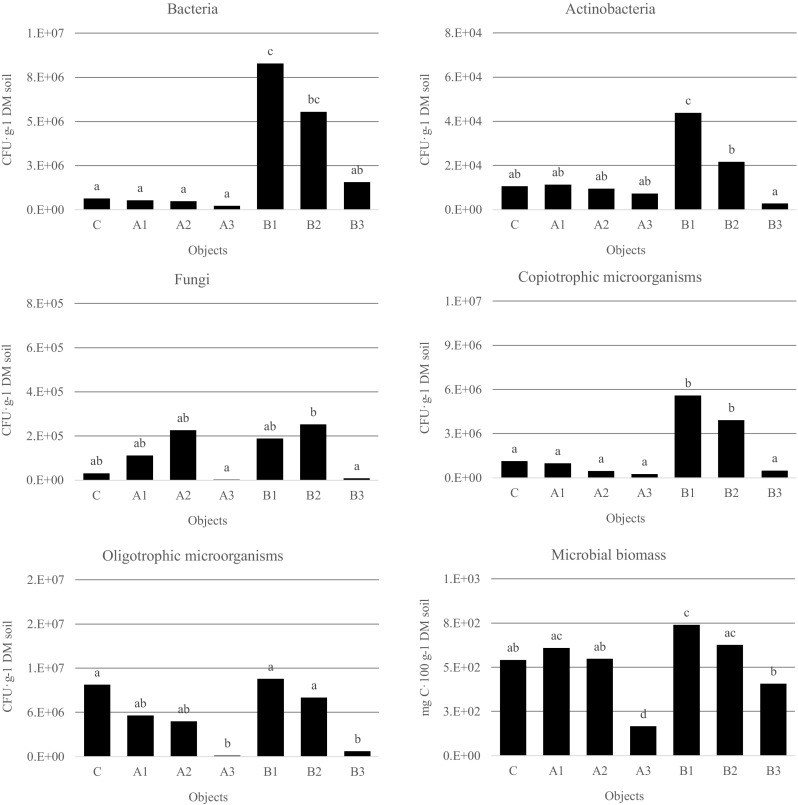
Average number and activity of microorganisms in individual objects of the experiment (mean over each columns not marked with the same letter is significantly different at *P* < 0.05)

Gasification of biomass is a promising technology of gaining renewable energy; however, waste created in the process including tar might be the cause of natural environment pollution (Mishra et al. 2015). In the evaluation of the effects of their influence, biological research is very helpful, which is a significant element of risk assessment connected with presence of toxic substances in particular elements of ecosystems. Based on chromatographic analysis, no significant differences in tar and its mixture with engine oil content were found; however, the reaction of microorganisms on presence of these pollutants depended not only on the group of microbes but also, in the first place, on the type and dose of pollution. In essential way, it could have been determined by different viscosity of both pollutants. The increase of CFU number, as well as reduction of their number, was observed. It was ascertained that introduction of tar and engine oil mixture (A) to soil caused decrease of number of bacteria, order of actinobacteria, oligotrophic, and copiotrophic microorganisms (Fig. 3). The amount of CFU decreased with the increase of pollution dose; however, significant differences were found only in the presence of the highest dose—10,000 mg kg^−1^ DM soil. The number of objects in soil polluted with tar (B) average CFU number was significantly higher than in objects in soil polluted with mixture of products (A). Besides fungi, the quantity of the remaining group of microorganisms in respective objects of the experiment was inversely proportional to the level of pollution. Ingredients of the waste, especially hydrocarbons, might be for some organisms a valuable source of carbon and energy, and then their number in the presence of this type of pollution might increase by many times in a short period of time, thus the observed significant stimulation of their growth. Differences in microorganisms’ reaction could have also resulted from the different chemical constitution of the examined liquid waste. The mixture contained engine oil, which consisted of, besides hydrocarbons, metalorganic ingredients (Butler and Mason 1997), and what is more significant amount of heavy metals appears in the used oil and the amount of polycyclic aromatic hydrocarbons (Keith and Telliard 1979) increases. Aromatic hydrocarbons may have slowed down microorganisms’ growth, and their toxicity grows with molecular mass growth, depending on configuration of benzene rings, substituent presence and position (Šepič et al. 1997). Disadvantageous influence of PAHs on soil microorganisms is confirmed by Hawrot-Paw and Ryłów (2011) research.

The value of coefficient O:C for individual objects of the research, which was determined on the base of the number of oligotrophic and copiotrophic microorganisms, contained within the range of 1.42 in soil polluted with tar dose 10,000 mg kg^−1^ DM soil to maximum 10.37 in the presence of mixture concentration 1000 mg kg^−1^ s DM soil. Superiority of copiotrophic over oligotrophic organisms (O:C = 0.45) was noted only in object A3, which can certify disorder in the stable level of organic matter in soil. Oligotrophic organisms often react unfavorably to carbon excess appearing in environment.

Content of microbial biomass is a good indicator of biological activity of the soil (Hawrot-Paw et al. 2010). Waste from the process of gasification in the dose 100 and 1000 mg kg^−1^ DM soil increased the amount of biomass in comparison to the not contaminated object (Fig. 3). Substantial increase, first of all, has been noticed in B1 object. The increase of the pollution level, in both kinds of waste, caused reduction of microorganisms’ activity.

The waste from gasification process had negative impact on biometric parameters of spring barley (Table 2). Conducted statistical analysis confirmed significant impact of the type of pollution and its dose on the height of the aboveground parts of plant as well as length of the root. Smith et al. (2006) examined the impact of the polycyclic hydrocarbons, including those present in the carbon tar, on seven species of grass and legumes. The authors did not ascertain the negative impact of hydrocarbons on germination; however, the presence of pollutants had unfavorable influence on further growth and development of plants, similar to the presented work. Negative impact on the observed results could also be the effect of heavy metals presence released to soil in the mixture of tar and fuel oil. These metals among others aluminum, chromium, cooper, iron, lead, manganese, and nickel, stopped by organic matter of soil, can be absorbed from soil solution by plant roots and cause chlorosis, leave spot disease, necrosis, and lowering of crops (Okonokhua et al. 2007; Adongbede and Sanni 2014).

**Table 2:** Results of biometrical measurements of spring barley

Objects	Biometric measurements [mm]	
	Lenght of shoots	Root length
C	190^a*^	163^a^
A1	154^ab^	109^b^
A2	39^c^	7^d^
A3	4^c^	0^d^
B1	189^a^	111^b^
B2	116^b^	41^cd^
B3	31^c^	8^d^

*Mean over each column not marked with the same letter is significantly different at *P* < 0.05

The applied mixture of tar and engine oil, dose of 10,000 mg kg^−1^ DM soil, showed toxic influence preventing growth of vegetative part of barley, so the determination of their physiological features was impossible. Statistical analysis showed that mixture of tar and engine oil (object A) used in the dose of 1000 mg kg^−1^ DM soil caused lowering of content of all kinds of assimilation pigments (chlorophyll “a”, chlorophyll “b”, total chlorophyll, carotenoids). In the case of total chlorophyll and carotenoids, the decrease was by 28% compared with the control (Table 3). Plants from this experimental combination (A2) were characterized with the lowest relative content of water in leaves tissues measured with RWC index. It has also been ascertained that tar pollution in the dose of 10,000 mg kg^−1^ DM soil had negative impact on synthesis of all kinds of assimilation pigments, causing lowering of their content in barley leaves from 35.8% in the case of carotenoids to 39.8% in the chlorophyll “b” case. Objects from the combination B3 were also characteristic for lower by 10%, compared to control, relative content of water. The research conducted by Hawrot-Paw et al. (2015) has proven the decrease of indicator of RWC in the leaves of pea seeds growing in the fuel oil pollution conditions.

**Table 3:** Results of physiological analysis of spring barley

Object	Chlorophyll a [mg g^−1^ fresh mass]	Chlorophyll b [mg g^−1^ fresh mass]	Chlorophyll total [mg g^−1^ fresh mass]	Carotenoids [mg g^−1^ fresh mass]	RWC [%]	T_FM_	F_V_/F_M_	PI	Area [bms]
C	1.231^b*^	0.708^b^	1.939^b^	2.192^b^	97.32^bc^	261.67^a^	0.804^bc^	1.355^b^	52,531.33^b^
A1	1.205^b^	0.687^b^	1.892^b^	2.234^b^	96.53^bc^	281.67^a^	0.803^bc^	1.337^b^	45,966.33^b^
A2	0.857^a^	0.527^a^	1.384^a^	1.579^a^	79.56^a^	248.33^a^	0.787^a^	0.930^a^	29,865.00^a^
A3	No data	No data	No data	No data	No data	No data	No data	No data	No data
B1	1.274^b^	0.732^b^	2.006^b^	2.314^b^	98.92^c^	266.67^a^	0.807^c^	1.423^b^	44,922.33^b^
B2	1.204^b^	0.697^b^	1.901^b^	2.155^b^	93.51^b^	253.33^a^	0.795^ab^	1.097^a^	35,522.83^a^
B3	0.767^a^	0.426^a^	1.193^a^	1.407^a^	87.52^a^	231.60^a^	0.740^a^	0.971^a^	35,566.3^a^

*Mean over each column not marked with the same letter is significantly different at *P* < 0.05

Metabolic process of plants especially sensitive to stress factors activity is photosynthesis. Measurement of chlorophyll “a” fluorescence allows, with high accuracy, to evaluate reaction of plants to photosynthesis process interference by stress factors as well as efficiency of reparation mechanism enabling keeping homeostasis despite unfavorable environmental conditions (Schapendonk et al. 1992, Lichtenthaler 1996, Spáčilová and Šafránkova 2011). Impact of both types of pollution applied in doses 1 and 2 on the value of TF_M_ that is on time of increase of chlorophyll fluorescence from the beginning of measuring to gaining maximum was not ascertained. Both types of pollutants applied in these doses lowered the value of system vitality indicator PSII (PI)—Table 3. In the case of polluting soil with tar, dose 10,000 mg kg^−1^ DM soil (B3), it was ascertained that it caused decrease of PI indicator decrease by 28.3% compared to control objects. The number of reduced plastoquinone electron acceptors (area) is one of the best indicators of assimilation apparatus efficiency measured with detection technique and analysis of fluorescence signal of chlorophyll “a.” In the conducted research, it was statistically proven that the used types of pollution in dose 1 as well as 2 lowered the value of this parameter, which according to Krause and Weiss (1991) and Kalaji and Łoboda (2007) proves the occurrence of stress phenomenon and blocking the transportation of electrons from reaction centers to plastoquinones. In the objects with combination B3, the value of area indicator was by 16.4% lower than the noted in control plants. The lowest value of F_V_/F_M_ parameter that is maximum potential efficiency of photochemical reaction in PSII (0.787) significantly different from the rest of experimental combinations except combination B2 (tar used in dose 1000 mg kg^−1^ DM soil) was ascertained in barley from combination A2 (mixture of tar and engine oil in the dose 1000 mg kg^−1^ DM soil). In the case of objects from the combination B3, the value was even smaller and amounted to 0.717. Maximum value F_V_/F_M_ achieved in the optimum conditions for plant development usually amounts 0.83. Its decrease certifies the occurrence of stress conditions.

## Conclusions

Significant influence on the size of the soil microorganism groups under investigation and their activity had the type of pollution as well as its dose. For most microorganisms, more unfavorable influence was observed in the presence of mixture of tar and engine oil than only tar itself. Number of microorganisms decreased along with the increase of soil pollution, while in object A, significant differences were observed in the first place in the presence of dose 10,000 mg kg^−1^ DM soil. This dose was also significant reduction factor in relation to microorganisms’ activity.

The examined impurities had unfavorable impact on growth and development of plants. Together with the increase of dose, independently of the type of pollution, decrease of height of the aboveground parts and length of roots of spring barley was observed. Taking into consideration the parameters of chlorophyll fluorescence induction, it can be ascertained that both types of pollution present in soil in the even smallest dose showed unfavorable influence on photosynthesis efficiency. In higher dose (1000 mg kg^−1^ DM soil), negative influence on synthesis of assimilation pigments and content of water in the plant had first of all the mixture of tar and engine oil (A). In the case of pollution applied in the highest concentration (10,000 mg kg^−1^ DM soil), higher toxicity for barley had the mixture of tar and engine oil.

Gasification of biomass, especially the waste, is one of the important methods of energy acquisition from renewable sources. Based on the analysis of acquired results, it was ascertained however that waste generated in this process might have unfavorable impact on the environment. It is advisable to undertake efforts aiming not only at limiting the amount of waste emerging from the technological process but also minimizing their abilities to penetrate the natural environment. In case of pollution, activities aiming at their removing should be undertaken because as it was shown their presence in soil, even in small concentration can negatively influence its biological life. Microorganisms and plants reaction might have been the result of direct toxic impact of pollution contents on microorganisms’ cells and plant tissues or might have been the effect of unfavorable physiochemical changes in soil, especially by mixture of tar and engine oil. Bioassays, in contrast to chemical analysis, showed significant differences between of both types of contamination. The selected tests could be useful not only for evaluating the toxicity of by-products but also for other applications, e.g., to assess the bioavailability of organic pollutants, the progress of remediation process.
